# How reliably can we predict the reliability of protein structure predictions?

**DOI:** 10.1186/1471-2105-9-137

**Published:** 2008-03-03

**Authors:** István Miklós, Ádám Novák ', Balázs Dombai, Jotun Hein

**Affiliations:** 1 Department of Statistics, University of Oxford, 1 South Parks Road, OX1 3TG Oxford, UK; 2 e-Science Regional Knowledge Centre, Eötvös Loránd University, Pázmány Péter sétány 1/a. 1117 Budapest, Hungary

## Abstract

**Background:**

Comparative methods have been the standard techniques for *in silico *protein structure prediction. The prediction is based on a multiple alignment that contains both reference sequences with known structures and the sequence whose unknown structure is predicted. Intensive research has been made to improve the quality of multiple alignments, since misaligned parts of the multiple alignment yield misleading predictions. However, sometimes all methods fail to predict the correct alignment, because the evolutionary signal is too weak to find the homologous parts due to the large number of mutations that separate the sequences.

**Results:**

Stochastic sequence alignment methods define a posterior distribution of possible multiple alignments. They can highlight the most likely alignment, and above that, they can give posterior probabilities for each alignment column. We made a comprehensive study on the HOMSTRAD database of structural alignments, predicting secondary structures in four different ways. We showed that alignment posterior probabilities correlate with the reliability of secondary structure predictions, though the strength of the correlation is different for different protocols. The correspondence between the reliability of secondary structure predictions and alignment posterior probabilities is the closest to the identity function when the secondary structure posterior probabilities are calculated from the posterior distribution of multiple alignments. The largest deviation from the identity function has been obtained in the case of predicting secondary structures from a single optimal pairwise alignment. We also showed that alignment posterior probabilities correlate with the 3D distances between *C*_*α *_amino acids in superimposed tertiary structures.

**Conclusion:**

Alignment posterior probabilities can be used to a *priori *detect errors in comparative models on the sequence alignment level.

## Background

Due to the increasing speed and number of genome sequencing projects, the gap between the number of known structures and the number of known protein sequences keeps increasing. As a result, demand for reliable computational methods today is higher than ever, while *in silico *estimation of protein structures remains one of the most challenging tasks in bioinformatics.

The central assumption of comparative bioinformatics methods for proteins is that the structures of proteins are more conserved than their amino-acid sequences. This allows homology modelling, namely, mapping the structure of a sequence onto homologous sequences. As insertions and deletions separating two homologous sequences accumulate, homologous characters in the two sequences will occupy different positions, which causes a non-trivial problem of identifying homologous positions. This problem can be solved by sequence alignment algorithms [1-4], which maximise the similarity between aligned positions while also minimise the insertions and deletions needed to align the sequences.

The relationship between gap-penalties and similarity scores can be set such that they maximise the number of correctly aligned positions in a benchmark set of alignments [5,6]. By contrast, stochastic models are capable to calibrate their parameters by applying a Maximum Likelihood approach even if no benchmark set is available. Hidden Markov Models were the first such stochastic models which have appeared in bioinformatics more than ten years ago [7]. Thorne, Kishino and Felsenstein introduced time-continuous Markov models for describing insertion and deletion events [8,9], and they showed on simulated data that the Maximum Likelihood method could correctly estimate the insertion-deletion as well as the substitution parameters with which the simulated data had been generated. The TKF models have subsequently been improved [10,11], and have been tested for alignment accuracy on biological data [11]. Above automatic parameter estimation, the other main advantage of stochastic models is that such models can provide posterior probabilities for each estimated alignment column as well as for the whole alignment, and these posterior probabilities correlate with the probability for the alignment column being correctly aligned [11-13].

The uncertainty in the sequence alignment can be slightly reduced when more than two sequences are simultaneously aligned together, and hence, much effort has been put in developing accurate multiple sequence alignment methods. Although efficient algorithms exist for any type of pairwise alignment problem, the multiple sequence alignment problem is hard. It has been proved that the optimal multiple sequence alignment problem under the sum-of-pairs scoring scheme is NP-hard [14], and it is strongly believed that the statistical approach to multiple sequence alignment is algorithmically not simpler than score-based approaches. Since it is unlikely that fast algorithms exist for any type of exact multiple sequence alignment problem, heuristic approaches have become widespread. Profile-HMM methods [15,16] align sequences to a profile-HMM instead of each other, and the multiple sequence alignment is obtained by aligning sequences together via a profile-HMM. Since the jumping and emission parameters of the HMM are learned from the data, this approach needs many sequences for parameter optimisation. Nevertheless, profile-HMMs do not consider evolutionary relationships amongst sequences, and hence, they cannot handle properly over-representation of evolutionary groups.

Iterative approaches have been introduced for score-based methods in the eighties [17,18] and have recently been extended for stochastic methods [13,19] using the transducer theory [20,21]. The drawback of iterative approaches is that in each iteration, they consider only a single, locally optimal alignment that might not lead to a globally optimal alignment. Moreover, as they consider only locally optimal partial solutions, they naturally underestimate the uncertainty of posterior probabilities.

The Markov chain Monte Carlo (MCMC) method represents a third way to attack the multiple stochastic alignment problem. It was first introduced for assessing the Bayesian distribution of evolutionary parameters of the TKF91 model aligning two sequences [22], and has subsequently been extended to multiple sequence alignment [23-28]. The general theory of Markov chain Monte Carlo [29,30] states that the Markov chain will be in the prescribed distribution after infinite number of random steps. Obviously, we cannot wait infinite many steps in practice, and therefore the success of MCMC methods depends on fast convergence: if the Markov chain converges quickly to the prescribed distribution, the bias of samples from the Markov chain after a limited number of steps will be negligible. The convergence can be checked by measuring autocorrelation in the log-likelihood trace or a few other statistics of the Markov chain and by running several parallel chains with different random starting points [31].

Since the above mentioned methods for multiple stochastic sequence alignment problems have been introduced only recently, no large-scale, comprehensive analysis on the performance of methods for protein structure prediction has been published yet. In this paper, we present a survey on how stochastic alignment methods can be used for protein secondary structure predictions. The prediction can be based on pairwise or multiple alignments and in both cases, either only a single, optimal alignment or the whole posterior distribution of alignments is used for prediction. We are interested in the question how much one can gain by involving more sequences and the posterior distribution of the alignments into the secondary structure prediction.

## Results

### Implementation of the methods

We implemented a stochastic pairwise and a stochastic multiple sequence alignment method in Java programming language (see Additional file 1), and we made a study of the methods on the HOMSTRAD database as described in the Methods section.

The stochastic pairwise alignment method was tested on all the possible 9494 pairs of sequences belonging to the same family. The analysis took two days on an Intel Xeon 3.0 GHz computer with SUSE Linux 9.3 operating system and JVM 1.5.0. The most time-consuming part of the analysis was the Maximum Likelihood parameter optimisation, which took approximately 90% of the total running time.

**Figure 1:**
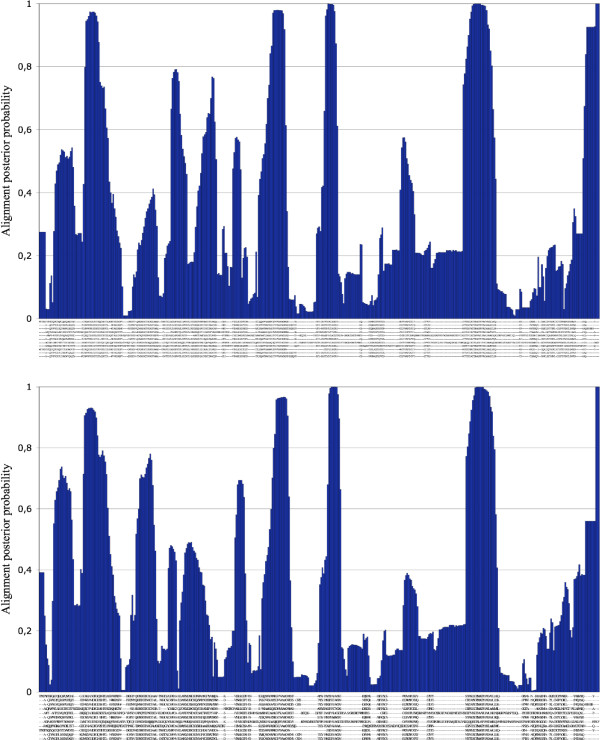
Maximum Posterior Decoding estimations for the multiple sequence alignment of the subtilase family in the HOMSTRAD database. The two estimations were given based on samples from two Markov chains with different starting points. The similarity between the two independent estimations shows good convergence and mixing of the Markov chain.

12 families have been selected for testing the stochastic multiple sequence alignment method, see Table 1. The families have been selected such that they reasonably cover the percentage identity distribtion of the HOMSTRAD database and they contain relatively many and approximately the same number of sequences. There are 541 possible pairs of homologous sequences obtainable from the 12 families, which is 5.7% of the possible homologous sequence pairs of the HOMSTRAD database. The analysis was performed on the ZUSE cluster of the Oxford Supercomputing Centre, each job ran on a dual Intel Xeon 3.6GHz processor under JVM 1.6.0. 1000000 MCMC steps were taken after convergence on each family. The running time of the analysis varied between 2.5 hours (7 sequences, length of 105 amino acids in average) and two days (11 sequences, 294 amino acids in average). The convergence has been verified based on the log-likelihood trace and comparing sampling distributions from parallel chains with different starting points, see Fig. 1. for an example.

**Table 1:** Selected families from the HOMSTRAD database for testing the performance of stochastic multiple sequence alignment methods

Family name	Class	Number of sequences	Average length	Average sequence id
Xylose isomerase	Alpha beta barrel	6	388	69%
Annexin	All alpha	6	317	57%
Calcium-binding protein – parvalbumin-like	All alpha	7	107	56%
Starch binding domain	All beta	8	105	52%
Glycosyl hydrolase family 22 (lysozyme)	Alpha+beta	12	126	51%
Legume lectin	All beta	12	234	50%
Papain family cysteine proteinase	Alpha+beta	13	223	40%
Subtilase	Alpha/beta	11	294	40%
Src homology 2 domains	Alpha+beta	11	105	35%
C-type lectin	Alpha+beta	8	126	27%
Halo-peroxidase	Alpha/beta	9	286	25%
Response regulator receiver domain	Alpha/beta	13	122	25%

### Post-processing the data

Secondary structure predictions have been given in four ways:

• Based on the Viterbi alignment (referred to as "Viterbi"). In this case, the most likely – a.k.a. Viterbi – alignment was obtained for all pairs of sequences and was used to map the secondary structure of one of the sequences onto the other sequence.

• Based on the posterior distribution of pairwise alignments using the Forward-Backward algorithm ("Forward"). In this case, the posterior probabilities that two amino acids are aligned together were obtained for all pairs of sequences and all pairs of amino acids. The secondary structure of one of the sequences was mapped onto the other sequence in a fuzzy way using the posterior probabilities.

• Based on the Maximum Posterior Decoding estimation from samples of a Markov chain Monte Carlo (MCMC) stochastic multiple alignment ("MPD"). In this case, the Maximum Posterior Decoding (MPD) alignments were predicted from MCMC samples and were used to map the secondary structure of one of the sequences onto the other sequences. The MPD alignment maximizes the product of the posterior probabilities of its alignment columns. See the Methods section for an explanation why the MPD alignment can be more accurately estimated from MCMC samples than the Viterbi alignment.

• Based on the posterior distribution of multiple alignments obtained by MCMC stochastic multiple alignment ("Bayesian"). In this case, the posterior probabilities that two amino acids are aligned together were estimated from the MCMC samples for all pair of sequences choosable from a multiple alignment and all pair of amino acids. The secondary structure of one of the sequences was mapped onto the other sequence in a fuzzy way using the posterior probabilities.

**Figure 2:**
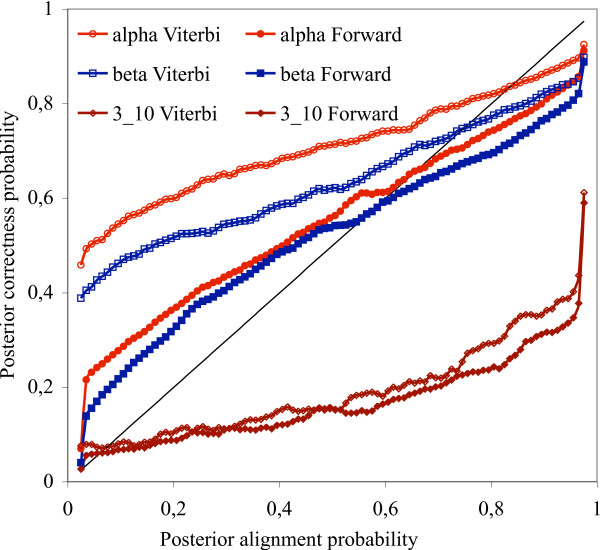
Posterior probabilities of correctly predicting secondary structure types with stochastic pairwise alignment methods as a function of alignment posterior probabilities. The black diagonal shows the identity function. The statistics have been generated on the whole HOMSTRAD database, 'Viterbi' means estimation based on a single, optimal alignment obtained by the Viterbi algorithm, 'Forward' means estimation based on the posterior distribution of alignments obtained by the Forward algorithm.

Amino acid sequences were divided into 100 categories based on their alignment posterior probabilities in the case of pairwise sequence alignments – or on their posterior structure prediction probabilities (see Methods, Eqn. 1.) in the case of Viterbi and Forward estimations, respectively. The 100 categories were evenly distributed on the [0, 1] interval. For each category and the three general types of secondary structures (alpha helices, beta sheets and 3_10 _helices), the percentage of the correctly estimated secondary structure types was calculated and plotted on Fig. 2. In the case of the Viterbi alignments, this means the ratio of the number of true positives and the number of all predictions of the given type. In the case of the Forward prediction, it is the number of amino acids of a given secondary structure type that fall in a particular category divided by the number of all amino acids in the category.

**Figure 3:**
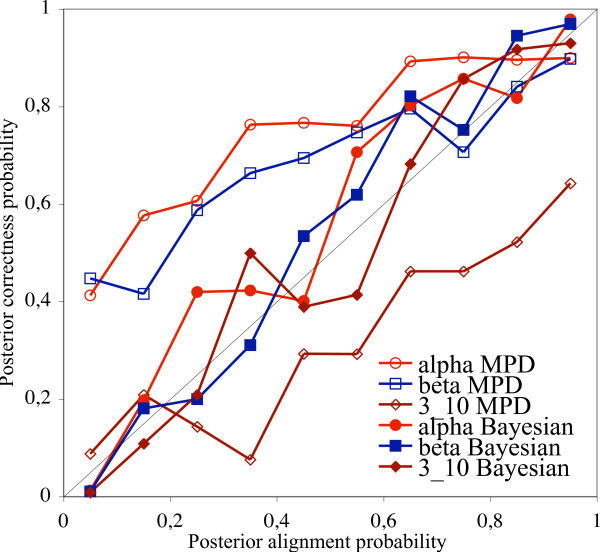
Posterior probabilities of correctly predicting secondary structure types with stochastic multiple sequence alignment methods as a function of alignment posterior probabilities. The black diagonal shows the identity function. The statistics have been generated on 12 families from the HOMSTRAD database, see Table 1.

Amino acid sequences of the selected 12 families were divided into 10 categories based on their alignment posterior probabilities in the case of multiple sequence alignments – or on their posterior structure prediction probabilities (see Methods, Eqn. 2.) in the case of MPD and Bayesian estimation, respectively. The 10 categories were evenly distributed on the [0, 1] interval. For each category and the three general types of secondary structures, the percentage of the correctly estimated secondary structure types was calculated and plotted on Fig. 3. In the case of the Maximum Posterior Decoding estimation, this means the ratio of the number of true positives and the number of all prediction of a given secondary structure type. In the case of Bayesian estimation, it is the number of amino acids of a given secondary structure type that fall in a particular category divided by the number of all amino acids in the category.

**Figure 4:**
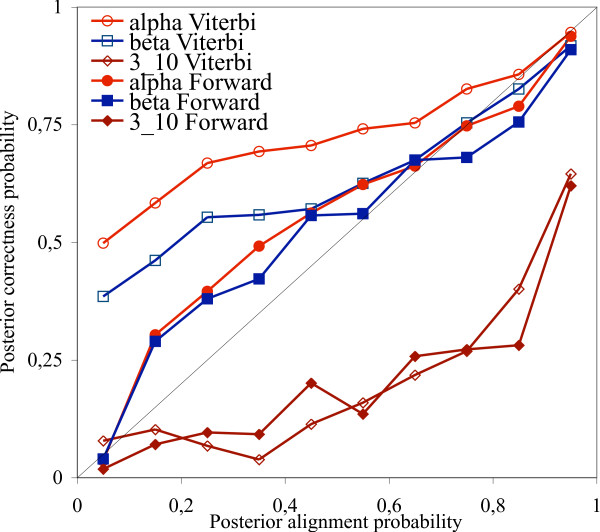
Posterior probabilities of correctly predicting secondary structure types with stochastic pairwise alignment methods as a function of alignment posterior probabilities. The black diagonal shows the identity function. The statistics have been generated on 12 families from the HOMSTRAD database, see Table 1.

For a fair comparison, we repeated the pairwise sequence comparison protocols on the selected 12 families, the generated statistics are shown on Fig. 4. The statistics obtained from the 12 selected families show similar behavior than those obtained from the whole HOMSTRAD database: all the six curves have similar shapes in both cases.

Our results indicate that methods predicting secondary structures based on a single alignment are over-pessimistic about their performance on alpha helices and beta sheets, namely, the posterior probabilities associated to the prediction are lower than the actual probability that the prediction is correct. Methods that predict structures based on the whole distribution of sequence alignments are less pessimistic – the alignment posterior probabilities better approximate the observed probabilities that the prediction is correct. All pairwise alignment methods proved to be over-optimistic estimating the reliability of their predictions for alpha helices and beta sheets with posterior probability above 0.8.

Predicting the correctness of 3_10 _helix predictions turned out to be the toughest of all secondary structure types. Each method except the Bayesian estimation on multiple sequence alignments is much over-optimistic on their power of predicting 3_10 _helices. MPD is less optimistic than pairwise methods.

Among all methods studied, Bayesian estimation based on multiple alignments was the only one that was able to correctly predict its prediction power of all secondary structure types, including 3_10 _helices, which makes MCMC-based multiple alignment methods successful candidates for promotion to a fundamental tool in protein structure prediction.

To show that the alignment posterior probabilities correlate not only with the goodness of secondary structure predictions but they also correlate with the similarities in the 3D structures, we calculated from the HOMSTRAD superimposed 3D structures the 3D distances between the *C*_*α *_atoms for each aligned pair of amino acids. The alignment posterior probabilities were evenly divided into 10 categories, and the average 3D distances as well as the low and high quartiles have been plotted for each category.

**Figure 5:**
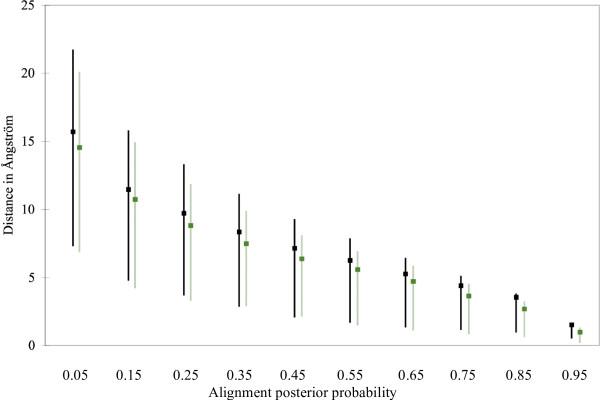
3D distances between the aligned *C*_*α *_amino acids as a function of pairwise alignment posterior probabilities. The 3D distances were calculated from the HOMSTRAD pdb files containing the superimposed structures of sequence families. Pairwise alignments were obtained by the Viterbi algorithm on the entire HOMSTRAD database (black) as well as on the 12 selected families described in Table 1. (light green). Boxes show the average distances, lines show the range between the low and high quartiles.

**Figure 6:**
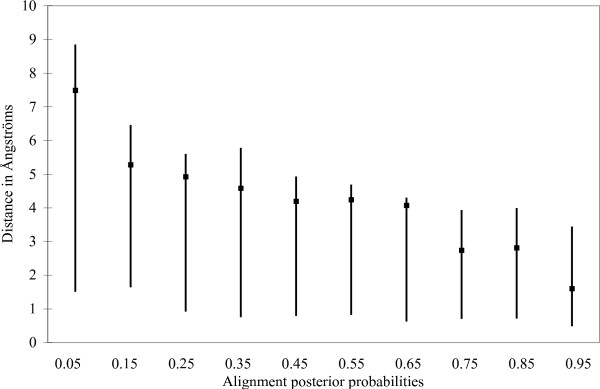
3D distances between the aligned *C*_*α *_amino acids as a function of multiple alignment posterior probabilities. The 3D distances were calculated from the HOMSTRAD pdb files containing the superimposed structures of sequence families. Multiple alignments are MPD estimations for the 12 selected families described in Table 1. based on MCMC samples. Boxes show the average distances, lines show the range between the low and high quartiles.

**Figure 7:**
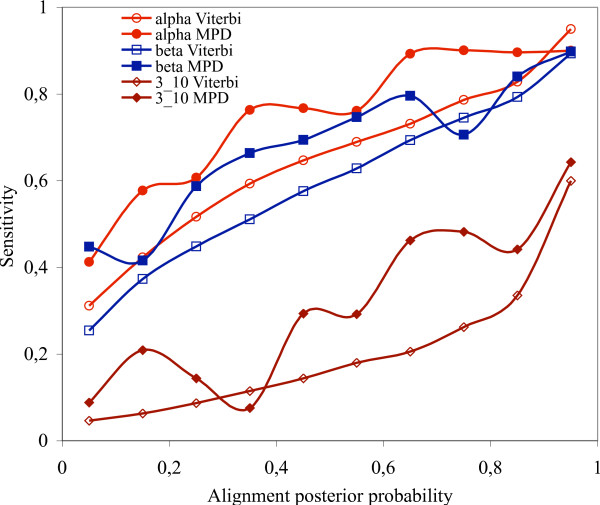
Sensitivity of secondary structure predictions as a function of alignment posterior probabilities. Sensitivity is defined as *TP*/(*TP *+ *FN*) where *TP *stands for the true positive estimations and *FN *stands for the false negative estimations. The Viterbi alignments were obtained for all possible homologous pairs in the HOMSTRAD database, the MPD alignments were estimated for the 12 selected families in Table 1.

Fig. 5. shows the results for Viterbi alignments, both for the entire database and for the 12 selected sequence families. Fig. 6. shows the results for the MPD alignments of the 12 selected families. Finally, we present on Fig. 7. the classical sensitivity values for the Viterbi and MPD alignments. Not only the posterior goodness probabilities correlate with the alignment posterior probabilities but also the sensitivity values. If secondary structure predictions are restricted for those alignment columns that have 0.8 alignment posterior probabilities or greater, then the sensitivity of alpha helix and beta-sheet predictions are greater than 80%, and about 50% for 3_10 _helices.

## Discussion

### Comparing predictions on different secondary structure types

The differences between the predictions of different secondary structure elements can be explained by their general attributes. Alpha helices are typically formed by 10 amino acids or more. Substitutions are frequent in alpha helices and they are surrounded by loop sequences where insertions and deletions often occur, therefore stochastic alignment methods realise some uncertainty, which yields relatively low posterior probabilities when aligning these regions. However, since alpha helices are relatively long, and the substitutions that occur in them rarely change the chemical behaviour of the affected amino acids, the long runs of chemically similar amino acids in the two sequences to be aligned give a strong statistical signal that helps align alpha helices.

Beta sheet elements are typically shorter than alpha helices, and are also surrounded by non-structured fragments accumulating insertions and deletions, which also yields relatively low alignment posterior probabilities. However, beta sheet elements are more likely to be misaligned, since their short length keeps them from carrying a statistical signal that alpha helices do.

**Figure 8:**
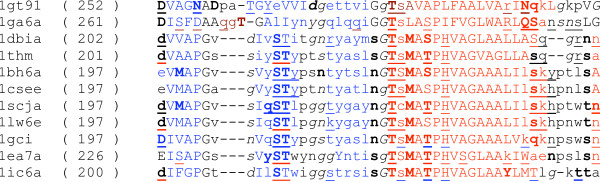
Part of the HOMSTRAD subtilase alignment in JOY format. In the middle of the alignment, the TSA motif might be both alpha helix and 3_10 _helix.

The 3_10 _helices are the least conserved secondary structure elements. Even if the actual amino acid sequence does not change, mutations at other parts of the sequence might indicate a conformation change that can shift the 3_10 _helix or transform it into a different structure type, see for example, Fig. 8. Such conserved parts are assigned high alignment posterior probabilities, and they increase the false positive ratio when this 3_10 _structure is mapped onto other sequences that do not contain this secondary structure motif. The fact that different secondary structure motifs can build up the same region of a functional protein implies that the given region might not be crucial to maintaining the structure and function of the protein and thus mutations can accumulate in the vicinity of the given region. Stochastic multiple sequence alignment can reveal the uncertainty in aligning that region, which explains why multiple alignment methods improve in predicting their predicting power on 3_10 _helices.

There is a similar explanation for the overoptimism in the region of 0.8 and higher posterior probabilities in the case of alpha helices and beta sheets: slight structural changes might shift the position where an alpha helix or a beta sheet starts or ends, even if the amino acids in the positions of question do not change. Fig. 8. also shows examples of such variations of secondary structure elements. For instance, the first alignment column is indicated to have a beta sheet structure in some sequences while it is non-structural in others.

### Comparing predictions of different protocols

Predictions based on a single, optimal pairwise or multiple alignment are over-pessimistic: alignment columns from both the Viterbi alignments and the MPD multiple alignments are labelled with posterior probabilities that are typically lower than the actual probability that the secondary structure predictions are correct for these columns. When the whole posterior ensemble of alignments is the basis of the secondary structure prediction, the posterior probabilities are closer to the actual probabilities that the prediction is correct. One main difference between the two strategies – prediction based on a single optimal alignment and prediction based on the posterior distribution of alignments – is that in the latter case posterior probabilities of all secondary structure types are given for each amino acid, while in the former case, the Viterbi or MPD alignment assigns at most one secondary structure element to each amino acid. This suggests the hypothesis that prediction methods based on the posterior distribution of alignments are less over-pessimistic due to possessing such false positive predictions with small posterior probabilities that are not part of a Viterbi or MPD alignment-based estimation.

To test this hypothesis, we predicted alpha helices and beta sheets from the posterior distribution of pairwise alignments in an alternative way. In this alternative prediction, each amino acid has been assigned to at most one secondary structure element that had maximal posterior probability (if the posterior probability of not harbouring a secondary structure type was maximal, then no secondary structure has been associated to the amino acid in question).

The correlation between alignment posterior probabilities and probabilities of correctly predicting a secondary structure type is obviously the same under the two different protocols if the posterior probability is greater than 0.5, since an event having probability greater than 0.5 must be the most likely event. The two types of curves split very soon below 0.5 (data not shown), and the second type of prediction protocol (considering at most one secondary structure type prediction for an amino acid) gets less over-pessimistic than the other protocol. This means that there are more true positive predictions than false positive predictions with non-maximal posterior probabilities.

**Figure 9:**
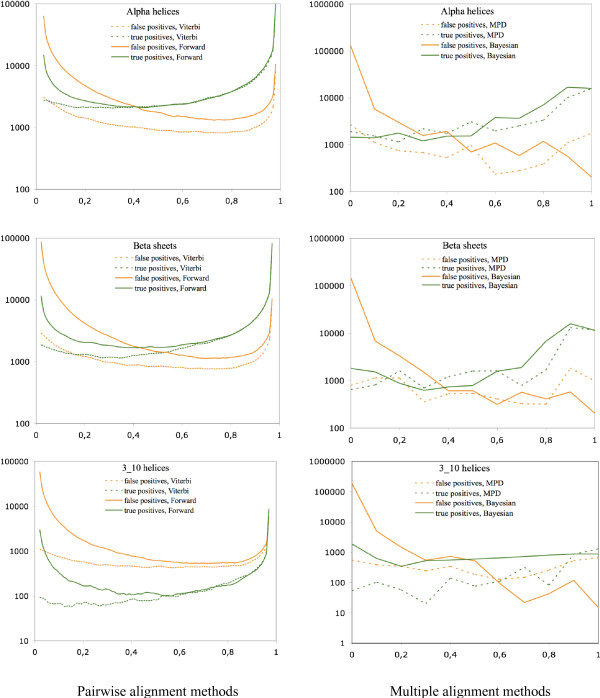
Number of true positive and false positive predictions as function of the alignment posterior probabilities.

This result is just the opposite of what our hypothesis suggested, therefore we also plotted the number of false positive and true positive predictions for each secondary structure type and prediction methods, see Fig. 9. This analysis confirms that explanation for methods based on the posterior distribution of alignments being able to predict their prediction power better than methods based on a single, optimal alignment is that they have more false positive predictions with alignment posterior probabilities below 0.5. The pairwise and multiple alignment methods show a different behaviour for alignment posterior probabilities greater than 0.5. While the Forward method has only slightly more true positive predictions and significantly more false positive predictions in this region than the Viterbi method, the Bayesian method has more true positive predictions and approximately the same false negative predictions as the Maximum Posterior Decoding method.

### Correlation between 3D structure similarities and alignment posterior probabilities

High alignment posterior probabilities indicate that the aligned residues are close to each other in the superimposed 3D structures. The average 3D distance between the aligned residues increases as the alignment posterior probability decreases. However, the distribution of residue distances become flatter for small alignment posterior probabilities, namely, a small alignment posterior probability does not necessarily mean that the aligned residues are far from each other. For example, 0.5 alignment posterior probability in a pairwise alignment means that there is still about 25% probability that the aligned residues are closer to each other than the average distance between amino acids that are aligned together with more than 0.9 posterior probability. The distance distribution is even flatter in case of multiple alignments. One possible explanation is that the alignment posterior probabilities are calculated for multiple alignment columns while distances are calculated for all possible pairs of amino acids in alignment columns. A small alignment posterior probability indicates possible differences in the 3D structures, however, some of the 3D structures might be still similar. Averaging the 3D distances in alignment columns naturally makes the distribution more centred (data not shown).

## Conclusion

In this paper, we studied how posterior probabilities of aligning characters in pairwise or multiple alignments might indicate whether secondary structure predictions based on the alignments in question are correct. We found that pairwise alignment methods are over-pessimistic on predicting alpha helices and beta sheets, namely, posterior probabilities of alignment columns are lower than the actual probability that the structure prediction based on the alignment column is correct, while they are overoptimistic on predicting 3_10 _helices, i.e., posterior probabilities for these alignment columns are greater than the probabilities that the secondary structure prediction for these amino acids is correct. Multiple alignment methods provide slightly more reliable predictions about their reliability of secondary structure predictions – they are less overoptimistic on 3_10 _helix predictions.

Secondary structure predictions can be given based on single, optimal pairwise or multiple alignments and also based on the posterior ensemble of alignments. In the latter case, posterior probabilities are closer to the probabilities that the secondary structure prediction is correct, especially when the structure prediction is based on the posterior distribution of multiple sequence alignments.

The multiple sequence alignment is the Holy Grail of bioinformatics [32] since what "one or two homologous sequences whisper ... a full multiple sequence alignment shouts out loud" [33]. Our experiments show that multiple sequence alignments not only highlight conserved positions better than pairwise alignments, but they also more reliably indicate the reliability of their prediction capabilities. This extra information could be exploited in 3D protein structure prediction: high posterior probabilities indicate the regions of the sequence alignment where the alignment accuracy is significantly better than the average alignment accuracy, see Figs. 5 and 6. These parts can be used as a reliable scaffold in homology modelling. On the remaining, unreliable parts, homology modelling is expected to have a low quality, and hence the 3D structure of these regions should be predicted with alternative methods, like *ab initio *threading methods [34-36].

It is worth mentioning that the alignment methods we applied in this work do not consider any information about how secondary structures evolve. It is well-known that different secondary structure elements follow different substitution processes, and this difference in the substitution pattern can be used for secondary structure prediction [37]. It is fairly straightforward to incorporate into current alignment methods *a priori *knowledge on the substitution, insertion and deletion processes of secondary structures, and we expect that such combined approaches will have a better performance in structure prediction. Nevertheless, secondary structures can be predicted not only in a comparative way, but also using a single sequence, based on the statistical properties of the amino acids in different secondary structure types [38,39]. Potential prior distributions for secondary types elements might be derived from such statistics and might be used in Bayesian analysis.

The running time of the methods obviously increases with the complexity of the background models, and analyses utilising such combined methods currently take too long to be applicable for everyday use on personal computers. However, the speed of processors keeps increasing exponentially following Moore's law, and will soon reach a level when it won't pose barrier to such combined approaches. Nevertheless, there are also promising channels to improve the running time of the methods. The standard approach for statistical multiple alignment is going to be MCMC, and current implementations make use of very basic tricks only, like the alignment window cut algorithm described in the Methods section. Several groups are working on making MCMC alignment methods more efficient and quickly mixing, and significant improvements are expected in the coming years.

## Methods

The HOMSTRAD database [40] has been downloaded and was used as a benchmark set for the methods we tested. As of December 2007, the database contains 1032 families of sequences, each family shares a common 3D structure. Each sequence in the database is annotated in JOY format [41] that, among other information, describes the secondary structure type of each amino acid (one of alpha helix, beta sheet, 3_10 _helix or none). We predicted the secondary structures of the sequences as described below.

### Pairwise sequence alignments

#### The stochastic model

**Figure 10:**
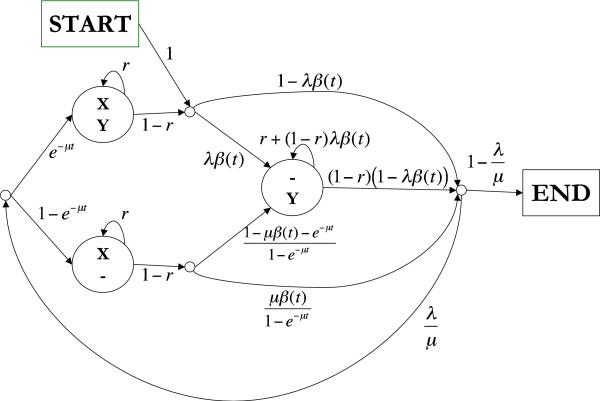
The TKF92 model [9], presented as a Hidden Markov Model. *λ *is the insertion rate, *μ *is the deletion rate, *r *is the parameter of the geometric distribution of inserted and deleted fragments, $\frac{1-e^{(\lambda -\mu )t}}{\mu -\lambda e^{(\lambda -\mu )t}}$. Emission probabilities are given by the time-continuous substitution model of the TKF92 model, the probability of joint emission of two characters equals the joint observation probability of two characters under the substitution model, single emissions follow the equilibrium distribution of the substitution process.

We used a simplified version of the TKF92 model [9] as presented in [11]. The simplified model can handle long insertions and deletions technically introduced as a birth-death process of unbreakable sequence fragments. Unlike the original TKF92 model, our model does not consider slowly and quickly evolving fragments, all fragments evolve with the same rate. Our model has a pair-HMM representation, see Fig. 10., in which a one-to-one correspondence between HMM paths and alignments exists such that the probability of any path in the pair-HMM equals the probability of the corresponding alignment in the modified TKF92 model. We used an optimised version of the Dayhoff rate matrix [42] for modelling the substitution process as described in [11,43].

#### Predicting secondary structure based on a single optimal alignment ("Viterbi")

For each family in the HOMSTRAD database, each pair of sequences has been aligned using the above described pair-HMM. Since the jumping probabilities in the pair-HMM are interdependent via common parameters, the usual EM algorithm [12] cannot be applied, instead, we made use of the conjugate gradient method [44] to get the numerical approximation of the Maximum Likelihood parameters for each pair of sequences. The Viterbi alignment [12] has been obtained for each sequence pair using the ML parameters, and for each alignment column in the Viterbi alignment, posterior probabilities have been calculated with the Forward and Backward algorithms [12]. The Viterbi alignment was used to map the secondary structure of one sequence to the other.

#### Predicting secondary structure based on the distribution of alignments ("Forward")

We also predicted secondary structures based on the distribution of alignments in the stochastic model. Posterior probabilities for each pair of characters from the two sequences have been obtained with the Forward and Backward algorithms using the Maximum Likelihood parameters. The posterior probability *P*_*s*_(*a*_*i*_) that a particular amino acid *a*_*i *_from sequence *A *has a secondary structure *s *given that it is related to sequence *B *is calculated as

(1) $$P_{s}(a_{i})=\sum_{j=1}^{m}P(a_{i},b_{j})\times \delta_{s,b_{j}}$$

where *m *is the length of sequence *B*, *P *(*a*_*i*_, *b*_*j*_) is the posterior probability that characters *a*_*i *_and *b*_*j *_are aligned, and $\delta_{s,b_{j}}$ is 1 if the known secondary structure of character *b*_*j *_is *s*, 0 otherwise.

### Multiple sequence alignment methods

#### Bayesian model for sequence alignments, evolutionary trees and model parameters

The transducer theory [20] has been used to construct a multiple-HMM along an evolutionary tree from pair-HMMs. The same pair-HMM described in the previous section was used in the construction, and the so-obtained multiple-HMM gives the likelihood of a multiple alignment and an evolutionary tree. This multiple-HMM describes sequence evolution as independent events on the branches of the evolutionary tree. This means that the sequence fragmentation on an edge of the evolutionary tree is not inherited on descending branches. Moreover, the fragmentations on sibling branches are independent from each other. Uninformative, exponential priors with expectation 1 have been used as priors for edge lengths and insertion-deletion parameters in the TKF92 model. All tree topologies were equally probable *a priori*. These priors together with the likelihood of a tree and multiple alignment on the tree define the joint posterior distribution of multiple sequence alignments, evolutionary trees and model parameters.

#### Markov chain Monte Carlo inferring of sequence alignments

Since the joint distribution of alignments, trees and parameters is a high dimensional distribution that is too complicated for direct, analytical inferring, Markov chain Monte Carlo [29,30] has been used for sampling from the posterior distribution. One of the key questions here is how far we can go with the analytical calculations. For the biologically less reliable, but computationally more tractable TKF91 model [8], we developed a fast algorithm [24,25] that calculates the likelihood of an evolutionary tree and a multiple sequence alignment of observed sequences. Such fast algorithm in the case of the TKF92 model is unknown, and hence, more data augmentation is necessary. This data augmentation includes sequences associated to the internal nodes and pairwise sequence alignments of neighbour nodes associated to the edges of the evolutionary tree. Since the likelihood of substitution events can be efficiently calculated with Felsenstein's algorithm [45], we only store the distribution of conditional likelihoods – also known as "Felsenstein's wildcards" [23] – at internal nodes of the evolutionary tree. We call this structure *extended alignment*.

The Markov chain performs a random walk on the space comprising the following components:

• Edge lengths of the tree

• Model parameters

• Extended alignment, described above

• Tree topology

We applied Metropolis-Hastings moves to change one of the components randomly, each component selected with a fixed, prescribed probability that was chosen to maximise the mixing of the Markov chain. Standard techniques were used for modifying edge lengths and parameters in the model, for a reference, see [25].

**Figure 11:**
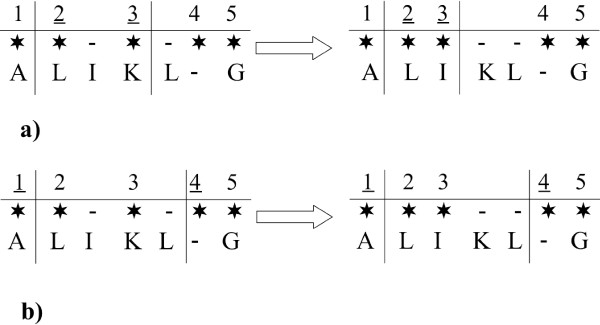
**a) **If the window borders are indicated by the first and last ancestral Felsenstein wildcard within the window (indicated as underlined), a proposed alignment could lead to a situation from which the original alignment could not be obtained by the same rules. **b) **If the window borders are indicated by neighbouring ancestral Felsenstein wildcards that are not within the window and will not to be realigned, no possible alignment will lead to such a situation, the original alignment will always be proposed back with a positive probability.

Changing the alignment is the most time-consuming event, since the running time of proposing a new alignment is proportional to the product of the lengths of the aligned sequences. A possible solution is modifying only a part of the alignment ("subalignment"), which decreases the running time of this type of proposal. Although it also decreases the mixing of the Markov chain, the overall performance of the Markov chain in terms of total computational time improves [22,25]. The subalignment is specified by a subtree and by the first and last column of the selected alignment region ("window") of the root node of this subtree. This window is extended to all nodes on the subtree, thus selecting a partial multiple alignment which would then be altered. However, since we have an extended alignment in the Markov chain, it is a non-trivial question how to propose a random subalignment in a way to maintain the reversibility of the move, which is required by the Metropolis-Hastings algorithm. The trick lies in the observation that if the borders of the selected window at the root node are marked with the neighbouring Felsenstein wildcards that *are not *within the window, then regardless of insertions or deletions at the beginning or end of the new alignment, the same window will be available for selection and this way the original alignment for (back)proposal. However, if the borders of the window were indicated by the first and last Felsenstein wildcards *within *the window, the proposal might not always be reversible – for an example, see Fig. 11 a). The distribution of window lengths is set such that the expected running time of an alignment changing step in the Markov chain grows approximately linearly with the lengths of the sequences.

**Figure 12:**
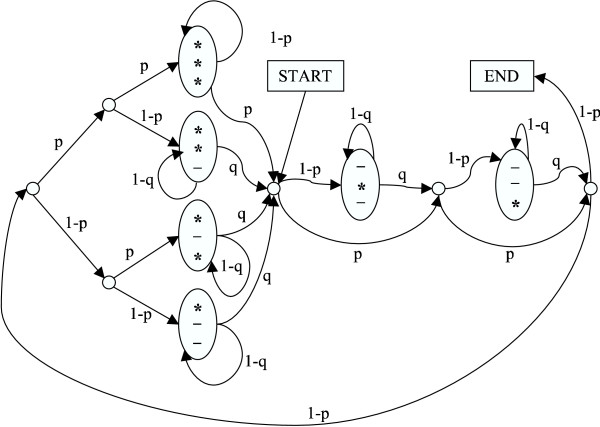
The pair-HMM that is used to realign sequences of the selected subtree. In all runs, *p *was set to 0.99 and *q *was set to 0.6. Emission probabilities followed the corresponding substitution model.

Sequences are iteratively realigned on the selected subtree within the selected window. In each iteration, the new alignment is drawn by the Forward-Backward sampling algorithm [12] with a pair-HMM with ancestral states ("HMM3"), see Fig. 12. We opted not to use the pair-HMM corresponding to the background model, since that would have seven non-silent states, while the model applied has only four after null-cycle elimination. This reduction of the number of states causes a speed boost of a factor of four to the calculation of proposal probability of the alignment change. The deviation from the TKF92 model did not cause low acceptance ratio for the alignment changing moves.

**Figure 13:**
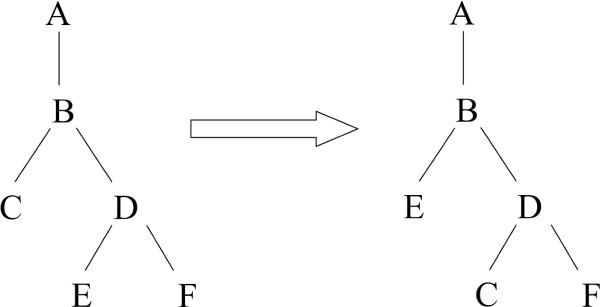
Effect of a single NNI step on a rooted subtree. A, C, E and F may or may not be leaf nodes.

We used nearest neighbour interchanges (NNI) for altering the topology as described in [46], which transform a rooted subtree in the way shown on Fig. 13. Since the alignment looses its validity after a topology change, the six affected sequences on the quartet are realigned after each nearest neighbour interchange move – the five pairwise alignments are obtained by first aligning C and F to D using the HMM3 shown above, then E and D to B the same way and last B to A using the pair-HMM.

Because the MCMC analysis is time-consuming, we selected 12 families from the HOMSTRAD database, see Table 1., on which we performed an MCMC analysis. The convergence was verified based on the log-likelihood trace and one million steps were taken in each Markov chain after its burn-in period. Each chain was sampled each 100 steps, so 10000 samples have been collected from each chain. In a few cases, alternative chains with different starting points were set up, and the MPD alignment has been estimated from both chains, see Fig. 1.

#### Predicting secondary structures based on the MPD estimation of multiple sequence alignment ("MPD")

In an earlier work [25], we showed that the maximum *a posteriori *(MAP) estimation from an MCMC sample is unstable, since there are many suboptimal alignments, and typically almost all sampled alignments from a Markov chain will be different. The same alignment showing up occasionally in multiple samples is merely due to the non-optimal mixing of the Markov chain, and such an alignment cannot be regarded as the most probable in the posterior distribution in any sense. Instead, we estimated the Maximum Posterior Decoding (MPD) alignment [12,47] that maximises the product of the posterior single-column probabilities. This method offers a significantly more reliable result since many alignments share particular columns. The estimation for the MPD alignment from an MCMC sample can be obtained by the simple dynamic programming algorithm which first creates a directed acyclic graph whose vertices are the alignment columns of the MCMC samples, and then estimates the posterior probability for each alignment column by the relative frequencies of alignment columns in the sample. The MPD estimation is the path that maximises the product of the relative frequencies. The MPD alignment is used to map the secondary structure of one of the sequences onto other sequences.

#### Predicting secondary structures based the posterior distribution of multiple alignments ("Bayesian")

We also predicted secondary structures based on all the alignments sampled from the Markov chain. The estimation for the posterior probability for a particular amino acid *a*_*i *_from sequence A having a secondary structure *s *given that it is related to sequence *B *is

(2) $$P_{s}(a_{i})=\frac{1}{N}\sum_{k=1}^{N}\delta_{s,f_{k}(a_{i})}$$

where *N *is the number of alignments in the Markov chain, *f*_*k*_(*a*_*i*_) is the amino acid in sequence *B *with which *a*_*i *_is aligned in the *k*th alignment, and *δ*_*s*,*x *_is 1 if the known secondary structure of character *x *is *s*, otherwise 0.

## Authors' contributions

IM proposed the research, contributed to the MCMC code and wrote some parts of the software for posterior analysis. AN wrote the majority of the MCMC code and the majority of the software for posterior processing. BD wrote the software for pairwise analysis. JH encouraged the research and wrote the manuscript.

## Supplementary Material

Additional file 1 Structure Projector package. Java source code and public licence in a   tar.gz archive Click here for file
